# Successful Treatment of AA Amyloidosis in Ankylosing Spondylitis Using Tocilizumab: Report of Two Cases and Review of the Literature

**DOI:** 10.3389/fmed.2021.661101

**Published:** 2021-04-26

**Authors:** Per Eriksson, Johan Mölne, Lina Wirestam, Christopher Sjöwall

**Affiliations:** ^1^Department of Biomedical and Clinical Sciences, Division of Inflammation and Infection, Linköping University, Linköping, Sweden; ^2^Department of Laboratory Medicine, Institute of Biomedicine, University of Gothenburg, Sahlgrenska Academy, Gothenburg, Sweden

**Keywords:** amyloidosis—diagnosis, ankylosing spondylitis, interleukin-6, nephropathy, proteinuria—nephrotic syndrome, tocilizumab

## Abstract

Historically, secondary amyloidosis has been a feared complication of chronic inflammatory conditions. The fibril protein AA derives from the acute phase reactant serum amyloid A (SAA). Long-term elevation of SAA levels remains a major risk factor for the development of AA amyloidosis in rheumatic diseases, and the prognosis may be unpredictable. Nowadays, with increased availability of effective biological agents, the incidence of AA amyloidosis seems to be declining. Still, genetically predisposed subjects with slowly progressive disease and mild symptoms combined with ongoing systemic inflammation may be at risk. Interleukin-6 (IL-6) is one of the drivers of SAA release and effectiveness of the humanized anti-IL-6 receptor antibody tocilizumab (TCZ) for the treatment of AA amyloidosis has been observed in some rheumatic conditions. Herein, we report two male subjects with longstanding ankylosing spondylitis (AS) complicated by renal amyloidosis who received TCZ with rapid and beneficial effects regarding inflammation and proteinuria. To the best of our knowledge, the use of TCZ in AS patients with this extra-articular manifestation has not previously been described. The paper includes histopathology, clinical follow-up, and longitudinal data of the two cases along with a comprehensive review of relevant literature. Mechanisms behind amyloid-mediated tissue damage and organ dysfunction are discussed. Altogether, our data highlight that blocking IL-6 signaling may represent a promising therapeutic option in patients with renal AA amyloidosis.

## Introduction

Systemic amyloidosis secondary to chronic inflammatory diseases (AA amyloidosis) was once a feared and common complication in rheumatoid arthritis (RA), ankylosing spondylitis (AS), inflammatory bowel disease (IBD), and autoinflammatory conditions (1). A large number of different types of amyloidosis exists, but the main subtypes are primary AL amyloidosis (light chains), secondary amyloid A (AA) amyloidosis, familial amyloidosis, and β_2_-microglobulin-related amyloidosis. The diagnosis of amyloidosis is based on clinical organ involvement and histological evidence of target organ showing deposition of abnormally folded proteins leading to organ dysfunction. Amyloid deposits are formed from globular, soluble proteins, which undergo misfolding and, subsequently, aggregate into insoluble fibrils or proteins may also have an intrinsic tendency to form amyloid in the absence of misfolding (2). Resistance to catabolism results in progressive tissue amyloid accumulation. Congo red is considered the gold standard dye owing to its higher sensitivity and specificity when differentiating amyloid from other protein deposits (3).

AA amyloidosis is characterized by the extracellular tissue deposition of fibrils that are composed of fragments of and/or intact serum amyloid A protein (SAA), a hepatic acute phase reactant (4, 5). Apart from the kidneys, which is the organ most commonly affected by systemic amyloidosis, involvement of the heart, the liver, the gastrointestinal tract and the peripheral nervous system should be considered. Thus, presence of proteinuria but also malabsorption, intestinal pseudo-obstruction, hepatomegaly, polyneuropathy, and restrictive myocarditis should raise the suspicion of AA amyloidosis in subjects with chronic inflammatory diseases (2).

The optimal treatment strategy of AA amyloidosis includes control of the underlying inflammatory disease and complete suppression of SAA production (5). Similarly to C-reactive protein (CRP), SAA constitutes an acute-phase reactant synthesized by hepatocytes but also by other cells, including macrophages, endothelial cells, and smooth muscle cells, under the transcriptional regulation of proinflammatory cytokines, particularly tumor necrosis factor (TNF) alpha, interleukin-1 (IL-1) beta, and IL-6 (4, 6, 7). Before the era of anti-cytokine targeted therapies, conventional synthetic Disease Modifying Anti-Rheumatic Drugs (DMARDs) such as azathioprine, chlorambucil, cyclophosphamide, and methotrexate were frequently used to avoid heavy proteinuria and subsequent renal failure in patients with AA amyloidosis (1). In recent years, TNF-blocking agents have been shown to reduce the risk of development of AA amyloidosis, as well as to improve the renal outcome of AA amyloidosis, in patients with inflammatory arthritides (8–11).

Although disruption of the tissue architecture is an established signum of amyloidsis, the histological and biochemical pathways leading to renal damage are less well-understood. Some observations indicate that amyloidogenic precursor proteins, folding intermediates and protofilaments possess toxicities that are independent of the amyloid deposits and that these toxicities contribute to organ damage (12). Furthermore, the amyloidogenic precursors appear to be toxic to cultured cells and tissues (13). In line with this, there is a lack of correlation between the quantity of amyloid in tissue and organ dysfunction (14, 15). However, divergent associations between the amount of amyloid deposits in kidney biopsy specimens and renal function have been reported (16–18).

Improved understanding of the mechanisms underlying amyloid deposition has enabled the development of new treatment strategies, specifically those targeting formation of amyloid proteins. As IL-6 is one of the important drivers of SAA release, it is conceivable to consider blocking of IL-6 signaling in AA amyloidosis (19). Indeed, effectiveness of the humanized anti-IL-6 receptor antibody (tocilizumab) for the treatment of AA amyloidosis in RA and juvenile idiopathic arthritis has been reported (20). However, to our knowledge, reports on IL-6 receptor blockade in patients with AS and secondary amyloidosis are scarce (21). Herein, we report two cases with longstanding AS who eventually developed AA amyloidosis and had a rapid renal improvement as a response to tocilizumab (TCZ). Informed consent was obtained from both patients.

## Clinical Cases

### First Clinical Case

The first subject is a male Kurdish–Iranian non-smoking patient who was diagnosed with AS in Tehran 1983 based on radiological findings and inflammatory back pain at the age of 28. After emigration to Sweden, he has been monitored at our unit since the age of 34. Despite slightly elevated levels of CRP, the AS gave only mild axial symptoms that only required continuous use of non-steroidal anti-inflammatory drugs (NSAIDs) and physiotherapy for many years combined with occasional use of glucocorticoids and analgesics during short periods. He acquired hypertension in his 50's, but the blood pressure is now well-controlled with metoprolol. At the age of 60, he was referred from his general practitioner to the hospital due to raised CRP and plasma creatinine (176 μmol/L), combined with low plasma albumin (22 g/L) and significant proteinuria (24-h protein excretion 0.52 g).

**Figure 1:**
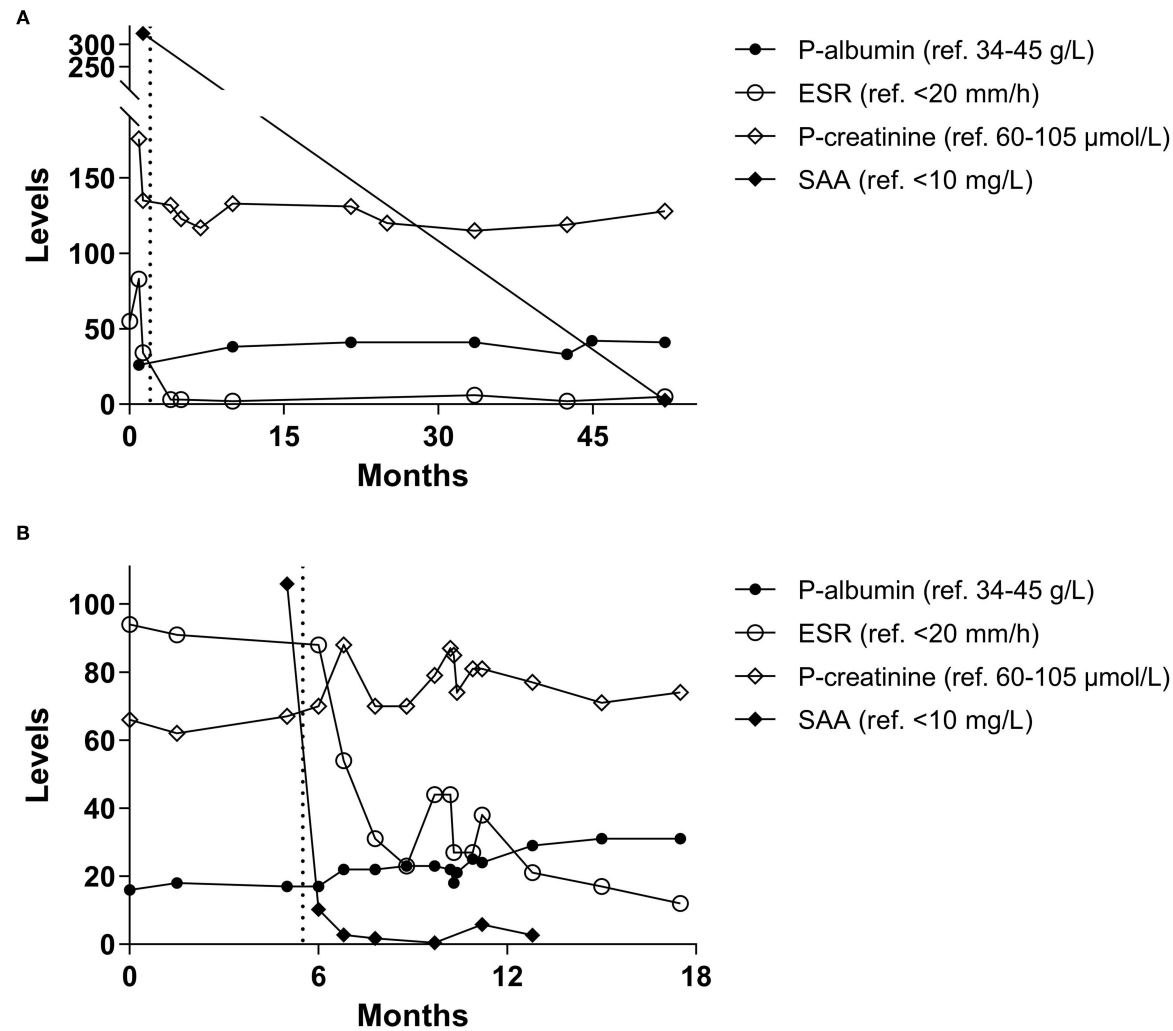
**(A)** Longitudinal laboratory data from the first clinical case with albumin and creatinine in plasma (P–), erythrocyte sedimentation rate (ESR), and serum amyloid A (SAA). **(B)** Longitudinal laboratory data from the second clinical case. Months indicate time since onset of proteinuria and the dotted line illustrates time-point of first tocilizumab administration.

Renal biopsy was performed. The histopathology was compatible with minimal glomerular amyloidosis stage 1A and interstitial amyloidosis, mainly in the medulla. Circulating levels of SAA were impressively high (325 mg/L). Infliximab (300 mg per infusion) was initiated without DMARD background, but the patient experienced an allergic reaction during the second infusion at our day care unit. Thereafter, monthly infusions of TCZ (480 mg per infusion, corresponding to 8 mg/kg) was started in monotherapy. After 3½ years, the TCZ infusions were replaced by weekly subcutaneous TCZ injections (162 mg) which are still ongoing without any reported side effects. Nevertheless, NSAIDs and analgesics are frequently required to manage the musculoskeletal symptoms. Figure 1A includes laboratory data of the first 52 months since the onset of proteinuria.

### Second Clinical Case

The second subject is a male Caucasian tobacco-smoking patient who was diagnosed with AS in 1984 at our unit. The diagnosis was based on radiological findings, although the inflammatory back pain had started already 1975 at the age of 34. During the 90's, he developed uveitis and Crohn's disease. The AS gave rather mild axial symptoms, but erythrocyte sedimentation rate (ESR) and CRP levels were constantly elevated. Whether the laboratory findings were attributed to the gut or the spine remained unclear. Sulfasalazine was tested during a short period but ceased early due to nausea. His IBD was treated with mesalazine and glucocorticoids. As he developed more symptoms from the spine combined with polymyalgia, subcutaneous injections with adalimumab 40 mg twice a month was added during a 6-month period but unfortunately without any relieve of musculoskeletal symptoms.

At the annual visit to rheumatologist in 2019, the patient presented with nephrotic syndrome based on proteinuria (urine albumin-to-creatinine ratio 913 g/mol; reference <3.0 g/mol) and low plasma albumin (16 g/L) whereas renal function apparently was preserved (estimated glomerular filtration rate based on plasma creatinine according to MDRD: >90 mL/min/1.73 m^2^). Renal histopathology was compatible with glomerular amyloidosis stage 1A and circulating levels of SAA were clearly elevated (106 mg/L).

Encouraged by our previous positive experience with IL-6 receptor blockade in AA amyloidosis, the patient was prescribed subcutaneous injections with TCZ (162 mg per week). Apart from an episode of fungal infection with esophagitis, which required hospitalization, TCZ has been well tolerated. The IBD had been quiescent for many years before the introduction of TCZ, precluding the possibility to judge any effect of TCZ on the intestine. However, the polymyalgia has almost disappeared according to the patient and no additional episodes of uveitis have been recorded. The patient is still prescribed TCZ weekly. Figure 1B includes laboratory data of the first 18 months since the onset of proteinuria.

### Histopathology

Renal biopsies were fixed in buffered paraformaldehyde, embedded in paraffin, sectioned, and stained with PAS, trichrome, htx-eosin, Ag-Jones, elastin/Van Gieson, and Congo red. Routine immunoperoxidase staining for IgG, IgA, IgM, C1q, C3c, C5b−9, and light chains were negative. Due to Congo red positivity, additional staining for SAA was performed. Table 1 summarizes and classifies the morphological findings and amyloid deposits according to Hopfer et al. (22).

**Table 1:** Histopathological analysis with scoring of amyloid deposition according to Hopfer et al. (22).

	**Case 1**	**Case 2**
Age	61	68
Glomeruli	5	14
• Global sclerosis (%)	2 (40)	3 (21)
• Amyloid (%)	5	10–40
• Stage	1	2
Interstitium		
• Interstitial amyloid (%)	40 (mainly medulla)	<1
• Fibrosis (%)	30	10
Vasculature	Transmural	Focal
Dominant pattern	Vascular and interstitial	Glomerular
Type	AA	AA

**Figure 2:**
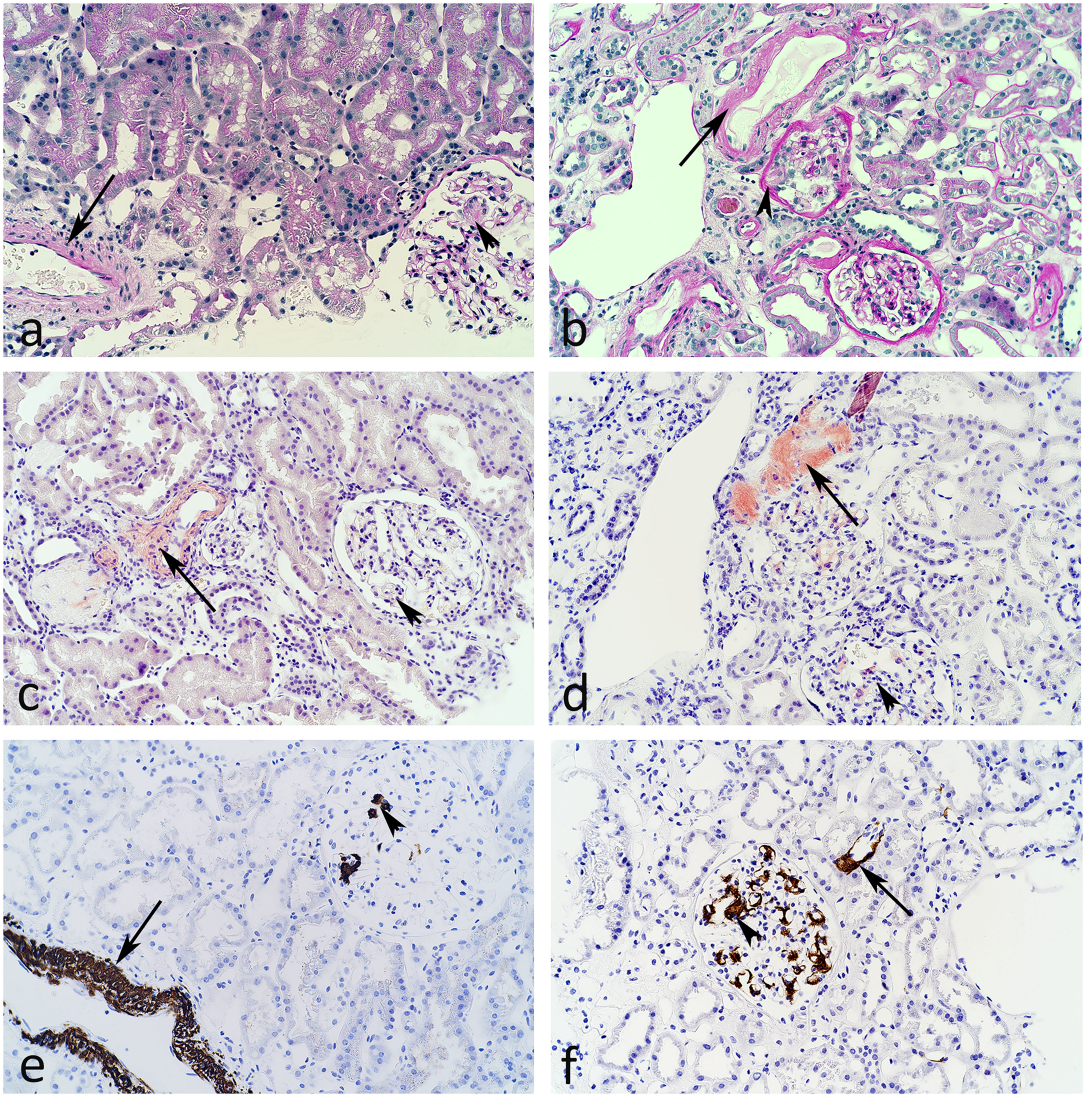
Light microscopic images from the first **(a,c,e)** and the second **(b,d,f)** clinical case described. PAS stain **(a,b)** indicates deposition of minimal amorphous material in arteries (arrows) and a glomerulus (arrowhead) in panel **(b)**. Congo red stain **(c,d)** reveal amyloid in arteries (arrows) and focally in glomeruli (arrowhead). In addition, polarized light showed birefringence. Using antibodies against serum amyloid A (SAA) and immunoperoxidase staining **(e,f)**, the amyloid is identified as of SAA-type in glomeruli, arterioli, arteries, and interstitium.

The biopsy from case 1 was sparse (11 mm) and contained mainly medulla. Two of five glomeruli were globally sclerosed and there were sparse, focal deposits of amyloid, mainly in the mesangium (Figure 2a). In the vessels, amyloid was found in both arteries and arterioli (Figure 2c). No amyloid was found in the cortex while large, focal amount of amyloid was found in the medulla (Figure 2a). SAA was positive in all Congo red deposits (Figure 2e).

The biopsy from case 2 was larger (total 23 mm) and contained mainly cortex. Three of fourteen glomeruli were globally sclerosed and glomeruli contained several foci of amyloid, mainly in the mesangium (Figure 2b). In the vessels, amyloid was detected in some but not all arterioles and arteries (Figure 2d). Limited amount of amyloid was observed in the interstitium.

**Figure 3:**
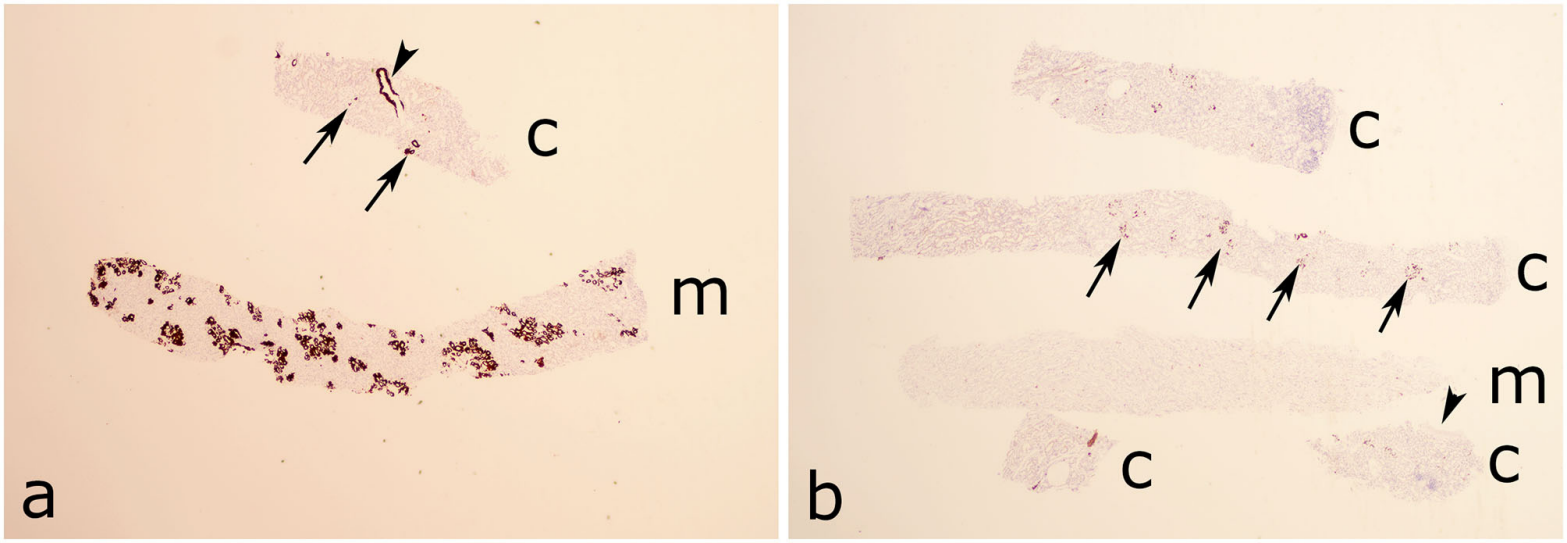
Overview micrograph with serum amyloid A (SAA) staining of the two biopsies. **(a)** In case 1 the amyloid is mainly localized in the medulla and focally in glomeruli and vessels. Cortex is sparse. **(b)** In case 2 the amyloid is focally distributed in glomeruli and negative in an artery and the interstitium. Arrows indicates glomeruli, arrowheads arteries. c, cortex; m, medulla.

SAA was positive in all Congo red deposits (Figure 2f) and an overview of the SAA positive staining is illustrated in Figure 3.

## Discussion

The last decade's widespread availability of highly efficacious biological agents for the treatment of rheumatological and other inflammatory disorders has not only resulted in fewer patients developing amyloidosis but also in improved overall survival (23, 24). Based on data from the Swedish Hospital Discharge Register and the Outpatients Register 2001–2008, AA amyloidosis among patients with RA had an estimated annual incidence of 2 per million (25). Yet, European data from the last two decades show that substantially younger individuals are nowadays diagnosed with AA amyloidosis, possibly indicating an increased awareness among clinicians (26, 27).

A nationwide, register-based study from Sweden demonstrated a prevalence of clinically diagnosed AS of 0.18% in 2009 with some phenotypical and treatment-related sex and socio-economic differences in disease prevalence (28). Reliable epidemiological data on AA amyloidosis in AS are more rare. A well-conducted study form Québec, Canada, concluded that the occurrence of renal amyloidosis in patients with AS was increased compared to the general population with a standardized prevalence ratio of 6.0 (95% confidence interval: 2.0–18.0). The data were particularly significant among men above the age of 60 (29).

A study from Turkey reported AA amyloidosis among ~1% of AS patients at a single referral center (30). Higher disease activity captured by the gold standard, self-reported, and validated instrument “Bath Ankylosing Spondylitis Disease Activity Index” (BASDAI) (31) in addition to higher age, longer duration of AS, elevated ESR, and the presence of peripheral arthritis were all associated with amyloidosis but only the initial BASDAI score remained as an independent predictor for the development of secondary amyloidosis in the multivariate analysis (30). As an historical illustration of the severity of this complication, data from Finland reveal that AA amyloidosis was an important cause of death in patients with AS before the era of biologics (32). Secondary amyloidosis was the immediate cause of death in 13% of all deaths among 398 cases with AS followed over almost 30 years at the Rheumatism Foundation Hospital in Heinola (32).

The two cases described herein fulfilled several of the risk factors identified in the Turkish study (30). At onset of proteinuria, they were above the age of 60 and had a long duration of AS. The disease had been slowly progressive and gave rather mild symptoms, which contrasted to the laboratory findings of systemic inflammation showing constantly elevated ESR and CRP levels. Their musculoskeletal symptoms had not been neglected at the annual visits to the rheumatology clinic, but a gradual adaptation of the patients to a decreased axial mobility cannot be excluded. Both patients had a short experience of TNF-blocking agents with inconclusive effects. Unfortunately, we were not able to retrieve initial BASDAI scores from any of them.

With increased availability of TNF inhibitors, the incidence of AA amyloidosis appears to be declining (4). As trials for IL-6 blocking agents like TCZ and sarilumab did not meet their primary endpoints in AS (33), the approach of initially adding a TNF-blocking agent in those AS patients who develop AA amyloidosis seems rational. Nevertheless, TCZ has been used with success in AA amyloidosis secondary to RA and familial Mediterranean fever (20, 34) and, to our knowledge, the two cases herein who received TCZ for AA amyloidosis secondary to AS are the first ones described. Neither did we find any report on the use of sarilumab in secondary amyloidosis related to any inflammatory disease. Besides directly IL-6 targeting agents, Janus kinase inhibitors reduce IL-6 signaling, and they may have impressive SAA-reducing effects in RA but none of the available drugs (tofacitinib, baricitinib, upadacitinib, and filgotinib) have consistently been evaluated in renal amyloidosis (35). However, of notice, upadacitinib is now licensed for the use in AS and renal impairment has a limited effect on upadacitinib pharmacokinetics (36, 37).

As demonstrated in Figure 1, both our patients had a rapid suppression of systemic inflammation combined with a reduction of proteinuria as a response to TCZ in monotherapy. None of the patients underwent a re-biopsy. Despite well-controlled systemic inflammation during TCZ therapy, the first clinical case witnessed increased axial symptoms that initially required addition of tenoxicam, and later etoricoxib. The second clinical case experienced relieve of musculoskeletal symptoms during TCZ and observed no deterioration of his IBD.

In conclusion, we report rapid and successful effects of TCZ in two male patients with longstanding AS complicated by renal amyloidosis. Inhibiting IL-6 is reasonable in AA amyloidosis but, to the best of our knowledge, experience of TCZ has not previously been published in patients with AS. These two clinical cases call for vigilance with regard to evolving proteinuria in AS patients with raised systemic inflammation, regardless of radiology and patient-reported symptoms.

## Author Contributions

CS had full access to all the data in the study and takes responsibility for the integrity of the data and the accuracy of the data analysis. PE, JM, LW, and CS: study conception and design and analysis and interpretation of data. PE, JM, and CS: acquisition of data. All authors were involved in drafting the article or revising it critically for important intellectual content, and all authors approved the final version to be published.

## Conflict of Interest

The authors declare that the research was conducted in the absence of any commercial or financial relationships that could be construed as a potential conflict of interest.
